# A randomised controlled pilot study: the effectiveness of narrative exposure therapy with adult survivors of the Sichuan earthquake

**DOI:** 10.1186/1471-244X-13-41

**Published:** 2013-01-31

**Authors:** Yinyin Zang, Nigel Hunt, Tom Cox

**Affiliations:** 1 Institute of Work, Health and Organisations, University of Nottingham, Nottingham, UK NG8 1BB; 2 School of Business, Economics & Informatics, Birkbeck University of London, London WC1, UK

**Keywords:** Earthquake, Exposure, Disaster, Narrative, PTSD, Therapy

## Abstract

**Background:**

Post-Traumatic Stress Disorder (PTSD) is a common psychological reaction after large-scale natural disasters. Given the number of people involved and shortage of resources in any major disaster, brief, pragmatic and easily trainable interventions are needed. The aim of this study is to evaluate the efficacy of Narrative Exposure Therapy (NET) as a short-term treatment for PTSD using Chinese earthquake survivors.

**Methods:**

A randomized waiting-list control pilot study was conducted between December 2009 and March 2010, at the site of the Sichuan earthquake in Beichuan County, China. Adult participants with newly diagnosed Post Traumatic Stress Disorder (PTSD) were randomly allocated to Narrative Exposure Therapy (NET) or a Waiting-List (WL) condition. The latter received NET treatment after a two-week waiting period. To compare the effectiveness of NET in traumatised earthquake survivors, both groups were assessed on PTSD symptoms, general mental health, anxiety and depression, social support, coping style and posttraumatic change before and after treatment and two months post treatment.

**Results:**

Adult participants (n=22) were randomly allocated to receive NET (n=11) or WL (n=11). Twenty two participants (11 in NET group, 11 in WL) were included in the analysis of primary outcomes. Compared with WL, NET showed significant reductions in PTSD symptoms, anxiety and depression, general mental stress and increased posttraumatic growth. The WL group later showed similar improvements after treatment. These changes remained stable for a two-month follow-up. Measures of social support and coping showed no stable effects.

**Conclusions:**

NET is effective in treating post-earthquake traumatic symptoms in adult Chinese earthquake survivors. The findings help advance current knowledge in the management of PTSD after natural disasters and inform future research. Larger sample sizes are needed to extend the present findings.

**Trial registration:**

Chinese Clinical Trial Registry ChiCTR-TRC-12002473

## Background

On May 12th, 2008 a devastating earthquake occurred in the Sichuan province of China. According to the Chinese Ministry of Civil Affairs, the earthquake destroyed almost 6.5 million homes and affected approximately 46 million people. Studies on earthquake victims suggest that Post Traumatic Stress Disorder (PTSD) and major depression are common psychological reactions [1-3]. Wang et al. [4]found that, three months after the Sichuan earthquake, the probable prevalence of PTSD was 37.8% in heavily affected communities and 13.0% in moderately affected communities. Similarly, Kun et al. [5] reported the prevalence of suspected PTSD was 45.5% in the heavily damaged Beichuan County and 9.4% in moderately damaged Langzhong County two and half months after the earthquake. Despite this evidence of high rates of PTSD in badly affected areas, no study has investigated the feasibility and effectiveness of psychological intervention with those affected.

Although PTSD therapies have been widely studied, most of them are in industrialized countries [6], such as the United States, Europe, or Australia. The context in earthquake populations in developing areas is different from the situation in industrialized areas. It is unclear to what extent knowledge about PTSD theory and treatment can be transferred to developing areas. In addition, most of the population in the serious damaged areas had been evacuated and were accommodated in temporary shelters or basic houses after the Sichuan earthquake. These survivors often continued to live in unstable living conditions, which were affected by serious floods in June 2008 and mud-rock flow on September 2008. People were also moved to unpredictable locations to facilitate the post-earthquake reconstruction. Previous studies have suggested that relocated individuals are more likely to experience psychological morbidity post-disaster (e.g. [7]). This may influence the effectiveness of psychological therapy. Furthermore, the gap between the many people in need of psychological assistance in the affected communities and the lack of qualified mental health professionals or counsellors is a major reason for the lack of scientific mental health provisioning after the earthquake. Studies regarding the effectiveness of simple, low-cost interventions are needed.

The most effective methods for the treatment of PTSD are trauma-focused approaches, and have been recommended over symptom-oriented psychotherapy [8]. Of the trauma-focused approaches, exposure and cognitive-behaviour therapy (CBT) have been shown to be effective in a range of from those with a history of sexual and physical assault to those having experienced accidents and natural disasters [9-11]. However, because these approaches are usually delivered in 10–12 sessions, they are still not sufficiently brief for use after large-scale disasters where there are large numbers of people who need assistance quickly, and there are likely to be few therapists available who are highly trained, as is required for CBT and exposure therapy. Furthermore, high dropout rates are reported in CBT treatment [12] showing that it is unsuitable for many people.

Narrative Exposure Therapy (NET; [13]) is a standardized short-term trauma-focused treatment approach developed to meet the needs of traumatised survivors of war and torture [14]. NET was developed based on principles derived from exposure therapy, CBT and testimony therapy. In contrast to other exposure treatments for PTSD, the patient does not identify a single traumatic event as a target in therapy. Instead, NET involves constructing a narrative that covers the patient’s entire life [15]. The cognitive processing model [16] asserts that PTSD symptoms are maintained through a distortion of explicit autobiographic memory about traumatic events and its detachment from the contents of implicit memory, which produces a fragmented narrative of the traumatic memories. Emotional processing theory [17,18] states that the habituation of emotional responses through exposure leads to a decrease in post-traumatic symptoms. Accordingly, NET stresses the importance of both approaches: the habituation of emotional responding to reminders of the traumatic event and the construction of a detailed narrative of the event and its consequences.

During NET, the participant constructs a detailed chronological account of his or her own biography in cooperation with the therapist. This autobiography is recorded by the therapist and corrected and improved with each subsequent reading. A special focus of the therapy is to integrate the generally fragmented, gap-filled reports of traumatic experience into a coherent narrative and to bring about the habituation of emotional responses to reminders of the traumatic event. During discussions of traumatic experiences, the therapist asks for current emotional, physiological, cognitive, and behavioural reactions and probes for relevant observations. The participant is encouraged to relive their emotions while reporting the events. The high level of emotional intensity around telling the event must have been attained and a notable reduction in fear and excitement must have been achieved before a session should be ended [14].

A review of NET [19] showed it to be effective for those with PTSD following multiple traumatic events such as those occurring in war or as a result of organised violence. Studies of NET in adults have consistently demonstrated its efficacy in treating individuals with PTSD living in a variety of low- and middle-income settings. One study [6] with Sudanese refugees in a Ugandan refugee camp showed NET had a better effect than support counselling and one-session psychological education. Another randomized controlled trial study with 277 participants with Rwandan and Somali refugees in Uganda [20] demonstrates how both mental health professionals and lay counsellors can deliver NET, and indicates the effectiveness of NET and relatively lower dropout rates compared with flexible trauma counselling. NET has not been applied to adult survivors of single natural disasters nor in Chinese settings.

Given the large number of people involved and the shortage of resources in any major disaster, any psychotherapeutic intervention must be simple, low-cost, quick and easy for local personnel to learn and use, even where there is little or no access to medical or psychological education. Furthermore, the method must be adaptable to the Chinese cultural environment. As oral narrative is common to all cultures, and previous studies have supported the effectiveness of NET in low and middle-income settings, it is likely that NET will be appropriate for Chinese earthquake survivors.

The goal of the present study was to examine whether adult survivors of the earthquake with a preliminary diagnosis of PTSD would profit from NET. The main measures examine PTSD symptoms, depression, anxiety, and general mental health. In order to gain a better understanding of the effectiveness of NET in this population, further individual factors that could contribute the effect of NET were also assessed, including social support, coping and posttraumatic growth.

Many studies have shown that social support is important in the development and maintenance of PTSD in diverse trauma populations [21], but little is known about the effect of interventions on social support. A longitudinal study conducted after a natural disaster revealed a reciprocal interaction between PTSD and social support [22]. Therapy may have a helpful effect on survivors’ perceived social support by reducing distress. The improved status may facilitate survivors’ reappraisal of the supportive efforts of others, and improve their ability to maintain a collective social network of usual and reliable acquaintances. Coping style is also important to understand the psychological consequences of traumatic events [23]. Previous studies have examined the reciprocal relationships between coping and PTSD symptoms [24]. Few studies have investigated coping style in a longitudinal setting and little is known about the effect of NET on coping. This study will also assess posttraumatic growth (PTG). Although people have reported perceived benefits following disaster [25], the impact of using NET on PTG is unknown.

It is hypothesised that NET will:

1. Significantly decrease symptoms of PTSD, depression and anxiety, and general mental health symptoms;

2. Significantly improve perceived social support and coping;

3. Lead to posttraumatic growth

## Methods

### Participants

The study used waiting list controlled, balanced randomization (1:1) and took place between December 2009 and March 2010 (19–23 months after the earthquake) in Beichuan County, an area severely affected by the earthquake. Participants were selected on the basis of scores on a screening programme within the framework of an ongoing mental and physical recovery programme. The screening was carried out among adults seeking assistance. Survivors who scored 20+ on the Impact of Event Scale-Revised (IES-R), which was part of the programme, were presented with the PTSD Diagnostic Scale (PDS) to confirm a diagnosis of PTSD. Eligible participants were all adults aged 18 or over who met the DSM-IV criteria of PTSD as measured by the PDS. Exclusion criteria included participation in another psychological treatment programme and an inability to finish the treatment due to relocation – several people changed their accommodation unpredictably and so were excluded.

Twenty two survivors were recruited to participate in this study. All participants gave informed consent after receiving a full explanation of the study design and objectives and explicit information regarding what the study entailed. This information was presented orally by a small research team of 3 researchers led by the first author. The study was approved by the Ethical Committee of the University of Nottingham.

### Measures

Severity of PTSD symptoms was assessed using the *Impact of Event Scale-Revised *(IES-R; [26]). This instrument is a self-report measure comprising 22 items and three subscales (intrusion, hyperarousal and avoidance), and scored on a 5-point Likert scale from not at all (0) to extremely (4). Cronbach alpha for the three subscales of the simplified Chinese IES-R have been reported as between 0.83-0.89 [27].Previous studies have provided good evidence that the simplified Chinese IES-R is a reliable and valid measure for assessing posttraumatic stress symptoms in a Chinese-speaking sample [28,29]. Structured interview instruments such as the Clinician-Administered PTSD Scale (CAPS), or the Structured Clinical Interview for DSM (SCID) were not available in Simplified Chinese language, and the IES-R is a time- and cost-efficient tool for obtaining information after large-scale disaster [27].

The *General Health Questionnaire-28* (GHQ-28; [30]) was used to assess the general mental health of the participants. This measure incorporates four subscales: somatic symptoms, anxiety and insomnia, social dysfunction, and severe depression. The simplified Chinese version of the GHQ-28 has been adopted widely and following its validation in Chinese [31] and with a reported Cronbach alpha of 0.92 with a sample of Chinese earthquake victims [32].

The *Short Form of the Changes in Outlook Questionnaire* (CiQQ-S; [25]) was used to assess both positive and negative posttraumatic changes. The 10-item CiQQ consists of a 5 items assessing positive changes, and 5 items assessing negative changes. Each item is answered on a 6-point scale ranging from strongly disagree (1) to strongly agree (6). The measure has been used in studies with a wide variety of participants following trauma and adversity [33]. The study of reliability and validity of Simplified Chinese version of CiOQ has been reported with a reported Cronbach alpha of 0.87 for positive change scale, and 0.82 for negative change scale [34].

Depression and anxiety were assessed using the *Hospital Anxiety and Depression Scale* (HADS; [35]), a widely used self-rating instrument for anxiety and depression.

The internal consistency, as assessed by Cronbach’s alpha, is 0.76 for the depression subscale and 0.79 for the anxiety subscale in a sample of Chinese hospital in-patients [36].

The *Multidimensional Scale of Perceived Social Support* (MSPSS [37]) was used to measure social support. The scale is designed to assess perceptions of the adequacy of social support from three different sources: family, friends, and significant others. It consists of 12 items; each item is scored using a 7-point Likert scale ranging from 1 (strongly disagree) to7 (strongly agree). Adequate reliability and validity have been reported for a simplified Chinese version [38], with a Cronbach alpha of 0.89.

The *Simplified Coping Style Questionnaire* (SCSQ [39] is a simplified Chinese instrument measuring two dimensions of coping style: active coping and passive. Active coping includes planning, thinking about solutions, and positive cognitive restructuring. Passive coping includes repression, behavioural disengagement, substance abuse and self-blame. There is a high correlation among the 12 items for active coping styles (Cronbach alpha=0.89) and the 8 items for passive coping styles. A Cronbach alpha of 0.78 was reported by Xie [39].

### Procedure

**Figure 1:**
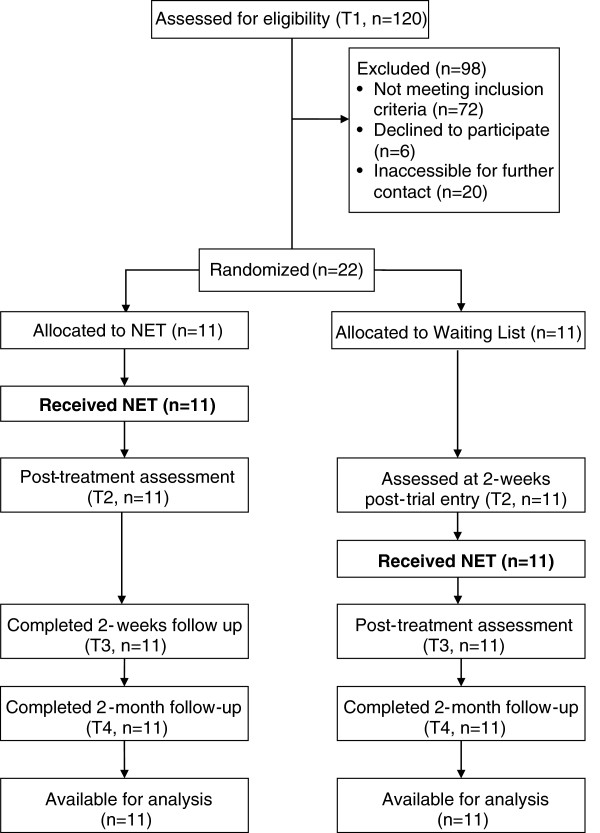
CONSORT diagram showing the flow of participants through each group.

Twenty two participants were randomly allocated to either NET (n=11) or a waiting list condition (WL; n=11) by a computer-generated list of random numbers. Those in the NET condition received therapy immediately; those in the WL condition received the same treatment after a waiting period. The assessment of screening process (T1) was used as the baseline. Those in the NET condition received 4 therapy sessions of 60–90 minutes each, which lasted 2 weeks with 2 ,3, or 4 days between each session, and were assessed post treatment (T2), after another 2 weeks (T3) and then after 2 months (T4) by using same scales. The WL controls were assessed 2 weeks after trial entry (waiting period) (T2), then given NET and assessed post treatment (T3) and finally assessed after 2-months (T4). Participants were informed that all scale items were focused on the earthquake as the trauma event to make sure that the latent psychological variables were associated with exposure to the earthquake. Figure 1 presents the research and treatment schedules for both conditions. There were no drop-out, with all participants completing the entire course of treatment and follow-up.

The screening process and treatments for both the NET and WL groups were carried out by the research team led by first author. The team was composed by three female therapists. All of them were native Chinese speakers, psychologists with Chinese national psychological counsellor certificate (Masters’ level) and were trained in the use of NET based on the manual [14]. Counsellors were closely tutored under supervision before beginning to work with clients. Case and personal supervision were maintained on a weekly basis. Treatment adherence was monitored by the direct observation of treatment sessions, by case discussions in supervision meetings, and by a review of the records and treatment protocols. The pre and post treatment assessments were carried out by a researcher not involved in treatment and blind to the treatment conditions. The details of the condition were unknown to the researcher. The two month follow up assessment was conducted by the first author over the telephone before data analysis. All scales were administered orally by interview as most participants were illiterate.

### Treatment

The treatment started shortly after the pretest. Four sessions of NET (90 minutes per session) were given to the participants as outlined in the manual. During these sessions, the patient, assisted by the therapist, constructed a detailed chronological report of his/her own biography with a special focus on the traumatic experiences. The narrative was recorded by the counsellor and corrected with each subsequent reading. The participants are encouraged to relive emotions while reporting the events. In the last session, the participant received a written report of his biography.

### Statistical analysis

Group differences in demographic data and pre-treatment measures were analysed by using chi-square tests and two-tailed t-tests. Pre- to post-treatment changes in questionnaire scores were analysed using univariate analyses of covariance (ANCOVAs), while controlling for pre-treatment scores. ANCOVAs are recommended as a robust and reliable statistical strategy for analysing the results of RCTs [40,41]. Within-group changes of each group from pre- to post- treatment were tested using paired t-tests. Hedge’s g was calculated as effect size for within- and between-group changes. The long-term treatment effect were analysed using repeated measures ANOVAs with the pre-test, post-test and follow-up scores and two groups. Pair wise differences were measured using paired t-tests with a Bonferroni correction. All analyses were performed in SPSS version 16.0.

## Results

### Treatment adherence

All participants constructed and completed a detailed chronological account of his own biography. The number of traumatic events they experienced was reported in Table 1. Participants’ reported previous traumatic experiences included difficult life conditions, family member’s terminal disease, or accidental injury. They did not report events such as violence, torture or persecution, events described in previous NET studies of refugees. Participants spent no more than one session on narrating previous traumatic events, with 2 to 3 sessions focused on the single incident of the earthquake. All participants completed the treatment. No major deviation from the study protocol was apparent.

**Table 1:** Sociodemographic characteristics of participants within the two treatment groups

	**NET (n=11)**	**WL (n=11)**	**Analysis**	
	**N**	**N**	***X***^***2***^	***p***
***Gender:***			0.00	0.99
Male	3	2		
Female	8	9		
***Marital status:***			0.00	0.99
Married	10	9		
Divorced or widowed	1	2		
***Education:***			1.01	0.59
Primary or below	8	9		
Junior middle school	2	2		
High School	1	0		
***Income:***			4.00	0.14
No fixed income	9	9		
Below £100	2	0		
£100-£300	0	2		
***Injured in the earthquake:***			0.79	0.66
Yes	5	3		
No	6	8		
***House damage***			1.07	0.59
Totally damaged	7	8		
Partically damaged	3	3		
Slightly damaged	1	0		
***Number of traumatic events experienced***			0.44	0.80
No	5	5		
2 or 3 times	4	5		
Over 3 times	2	1		
***Type of traumatic events***				
Difficult life condition	9	10		
Accidental injure	4	2		
Family number’s disease or loss	2	2		
**Age**	M(SD)	M(SD)	*t*	*p*
	56.64(12.22)	54.82(11.59)	0.36	0.730

### Baseline data

The age range of the sample was 37 to 75 (55.7 ±11.7). The socio demographic characteristics of the participants are described in Table 1. All were of low socio-economic status. There were no significant differences between the two groups regarding age, gender, education, marital status, income, injuries, and house damage.

### Treatment effect

Table 2 showed the mean scale scores of two groups at each time point (T1, T2, T3, and T4). At baseline (T1), there is no significant difference between two groups.

**Table 2:** Measures over time for treatment and waitlist control group

	**T1**		**T2**		**T3**		**T4**	
**Measures**	***Mean***	***SD***	***Mean***	***SD***	***Mean***	***SD***	***Mean***	***SD***
**IES-R**								
**Avoidence**								
*NET*	17.64	9.19	9.09	5.43	8.45	5.22	9.73	6.20
*WL*	15.73	4.08	16.09	3.65	9.36	2.98	8.46	3.30
**Intrusion**								
*NET*	16.91	6.88	8.73	4.56	8.36	3.91	7.73	5.10
*WL*	17.36	3.17	16.00	4.29	9.00	3.55	8.55	2.77
**Hyperarousal**								
*NET*	13.64	4.48	8.00	3.82	7.72	4.13	6.64	4.39
*WL*	14.55	3.59	15.09	2.98	9.91	2.88	8.55	2.54
**GHQ-28**								
*NET*	9.45	5.05	3.00	3.38	2.82	3.63	2.73	3.35
*WL*	13.27	6.94	13.64	5.75	5.55	3.45	5.09	2.70
**HADS**								
**Anxiety**								
*NET*	8.45	3.86	5.27	2.83	5.00	2.53	5.45	3.03
*WL*	9.55	4.91	8.64	3.56	5.00	3.16	4.82	2.48
**Depression**								
*NET*	8.18	4.21	4.18	2.36	5.00	2.68	4.91	3.02
*WL*	7.09	3.21	7.09	2.95	4.00	2.24	3.73	2.10
**CiOQ**								
**Positive**								
*NET*	24.82	3.66	26.91	2.43	27.45	2.11	28.00	1.79
*WL*	26.82	2.27	26.73	2.33	28.27	2.24	28.09	2.12
**Negative**								
*NET*	16.55	7.92	11.82	5.78	11.09	6.07	10.64	4.25
*WL*	15.00	7.25	15.82	6.84	11.36	5.82	9.90	3.18
**MSPSS**								
*NET*	60.64	13.06	62.00	11.57	60.64	10.86	61.27	11.72
*WL*	61.00	6.96	58.91	5.77	60.18	9.38	57.55	5.50
**SCSQ**								
**Active**								
*NET*	23.45	7.93	25.09	6.77	23.45	6.07	23.82	8.57
*WL*	24.18	5.96	23.09	5.39	24.73	4.08	24.18	5.10
**Passive**								
*NET*	11.18	4.29	10.27	3.23	10.45	3.45	10.09	2.84
*WL*	11.45	2.58	11.73	2.97	10.82	2.96	10.45	1.57

### Initial treatment outcome

The initial treatment outcome analyses are described in Table 3. Paired t-tests revealed there were no significant within-group changes in the scores for WL group across its waiting period but there were significant within-group changes in the scores of the NET group with treatment on avoidance, intrusion, hyperarousal, GHQ-28, anxiety and depression, positive and negative changes.

**Table 3:** Results of outcome measures of T1 and T2

**Measures**	**Groups**	**Mean difference (T1-T2)**		**Within-groups**		**Effect size**	**Between-groups**		**Effect size**
			**95% CI**	***df***	***t***		***df***	***F***	
**IES-R Avoidance**	*NET*	8.55	(3.85 to 13.24)	10	4.05**	1.09	1,19	28.99***	1.46
	*WL*	−0.36	(1.54 to 0.81)	10	−0.69	0.09			
**IES-R Intrusion**	*NET*	8.18	(4.40 to 11.97)	10	4.82**	1.35	1,19	22.20***	1.58
	*WL*	1.36	(−0.02 to 2.75)	10	2.19	0.35			
**IES-R Hyperarousal**	*NET*	5.64	(4.04 to 7.23)	10	7.68***	1.30	1,19	57.30***	1.99
	*WL*	−0.55	(−2.02 to 0.94)	10	−0.82	0.16			
**GHQ-28**	*NET*	6.45	(4.72 to 8.19)	10	8.29***	1.44	1,19	33.33***	2.17
	*WL*	−0.36	(−3.85 to 3.11)	10	−0.23	0.06			
**HADS Anxiety**	*NET*	3.18	(2.06 to 4.30)	10	6.35***	0.90	1,19	21.38***	1.01
	*WL*	0.91	(−0.58 to 2.40)	10	1.36	0.20			
**HADS Depression**	*NET*	4.00	(1.83 to 6.17)	10	4.11**	1.13	1,19	14.57**	1.05
	*WL*	0.00	(−1.62 to 1.62)	10	0.00	0.00			
**CiOQ Positive**	*NET*	−2.09	(−3.19 to −0.99)	10	4.23**	0.65	1,19	3.86^a^	0.07
	*WL*	0.09	(−1.39 to 1.58)	10	0.14	0.04			
**CiOQ Negative**	*NET*	4.73	(2.50 to 6.96)	10	4.72**	0.66	1,19	32.14***	0.61
	*WL*	−0.82	(−1.97 to 0.34)	10	−1.58	0.12			
**MSPSS**	*NET*	−1.36	(−4.20 to 1.48)	10	1.07	0.11	1,19	4.25^b^	0.33
	*WL*	2.09	(−0.86 to 5.04)	10	1.60	0.31			
**SCSQ Active**	*NET*	−1.64	(−3.85 to 0.58)	10	−1.65	0.21	1,19	5.21*	0.31
	*WL*	2.59	(−0.65 to2.83)	10	1.40	0.18			
**SCSQ Passive**	*NET*	0.91	(−0.38 to 2.20)	10	1.57	0.23	1,19	4.44*	0.45
	*WL*	−0.27	(−0.95 to 0.41)	10	−0.90	0.10			

Means difference, 95% CI, paired t-test, within group effect sizes, ANCOVA analysis, and between group effect sizes.

*p<0.05; **p<0.01; ***p<0.001.

^a.^*p=0.06.*

^b*.*^*p=0.05.*

Univariate ANCOVAs on post- treatment scores controlling for pre- treatment scores revealed significant effects for IES-R, GHQ-28, HADS, CiOQ and SCSQ. Following the treatment at the waiting period (T2), there were significant differences between the scores of the NET and WL groups on avoidance, intrusion, hyperarousal, GHQ-28, anxiety and depression, negative changes, active coping, passive coping, and trends towards higher on social support *(p*=0.06*)* and positive changes *(p*=0.05*).*

Within- and between-group effect sizes for the outcome measures are included in Tables 3. From pre to post treatment, large (≥.80) within-group effect sizes were found for the Treatment group on the avoidance, intrusion, hyperarousal, GHQ-28, anxiety and depression, moderate (.50-.79) within-group effects were found on the positive and negative changes. Large between- group effect sizes were found on the avoidance, intrusion, hyperarousal, GHQ-28, anxiety and depression, moderate between-group effect sizes were found on the negative changes, and small (.20-.49) between-group effect sizes were found on active and passive coping.

### Two-month follow-up outcome

As the WL group received the treatment after T2, the scores of T2 for WL group were taken as their pre-test baseline. The pre-test, post-test and 2-month follow-up scores were analysed using repeated measures ANOVAs with two groups. Table 4 presents the repeated measures ANOVAs with three levels of time: pre-treatment (T1 for NET and T2 for WL), post treatment (T2 for NET and T3 for WL) and at 2 month follow up (T4 for both groups) and treatment group (NET vs. WL) as between-subjects variable.

**Table 4:** Repeated ANOVA of time (pre-treatment, post-treatment, follow-up) × group (NET, WL) with post-hoc Bonferroni tests

	**Time**		**Time × group**		***pre/post***	***pre/fu***	***post/fu***
**Measures**	***df***	***F***	***df***	***F***	***p***	***p***	***p***
IES-R Avoidance	2,19	23.83***	2,19	1.22	***	***	-
IES-R Intrusion	2,19	25.28***	2,19	0.35	***	***	-
IES-R Hyperarousal	2,19	58.24***	2,19	0.09	***	***	-
GHQ-28	2,19	41.79***	2,19	0.59	***	***	-
HADS Anxiety	2,19	44.96***	2,19	0.67	***	***	-
HADS Depression	2,19	23.35***	2,19	0.69	***	***	-
CiOQ Positive	2,19	9.53**	2,19	1.04	***	*	-
CiOQ Negative	2,19	22.85***	2,19	0.04	***	***	-
MSPSS	2,19	0.85	2,19	0.15	-	-	-
SCSQ Active	2,19	2.13	2,19	0.11	-	-	-
SCSQ Passive	2,19	3.00	2,19	0.03	-	-	-

*p<0.05; **p<0.01; ***p<0.001.

**Figure 2:**
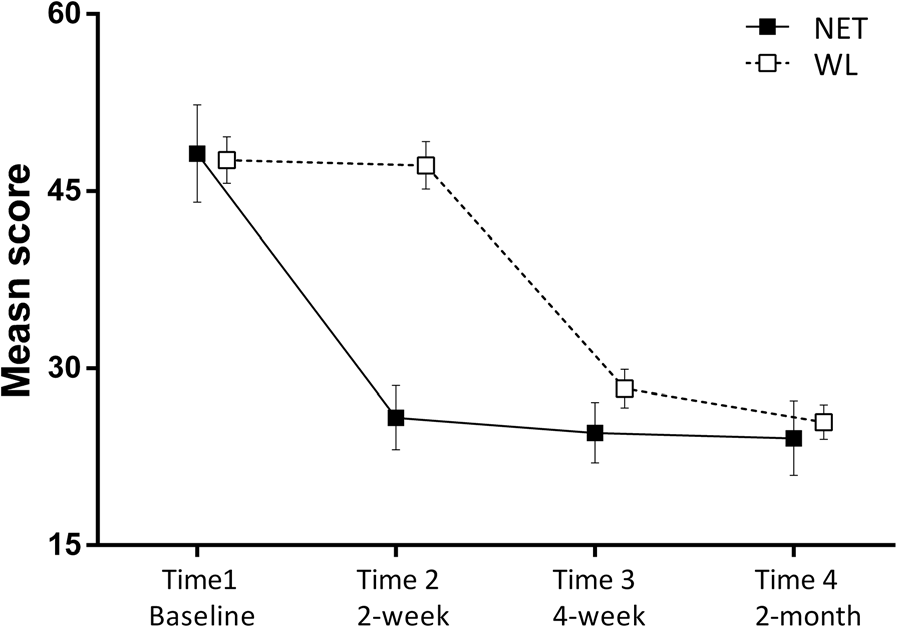
**Mean scores for IES-R of two groups. **The WL group did not undergo NET during the first 2 week of the study. At Time 2, participants in the NET had significantly lower self-reported symptoms of PTSD than did participants in the WL group. At Time3, after the WL group completed the NET treatment, a difference no longer existed between the groups. The effect maintained in Time 4 (2-month follow-up). Error bars indicate standard errors.

**Figure 3:**
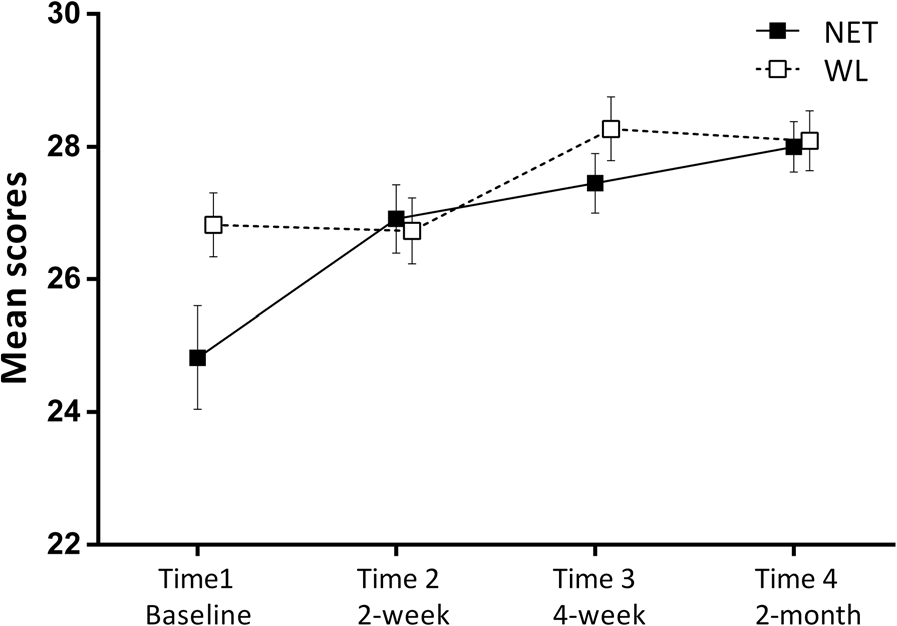
**Mean scores for positive changes of two groups. **At Time 2, after the NET group underwent the treatment, their positive change score was significantly higher than the score at Time1, and there was no change for WL group. At Time3, after the WL group completed the treatment, their positive score increased significantly. The effect of two groups maintained in Time 4 (2-month follow-up). Error bars indicate standard errors.

There were significant time effects post-treatment for the measures of IES-R, GHQ-28, HADS and CiQQ. There were no significant time × group interaction effects for any of the measures. Comparison of pre-, post-, and follow-up showed a significant reduction of scores after treatment in IES-R, GHQ, HADS, CIOQ, with these changes remaining stable at the 2 month follow-up. These indicated that, for both groups, overall PTSD symptoms across the three PTSD symptom clusters (intrusion, avoidance, and hyper arousal) (Figure 2), general mental health, depression and anxiety, and negative changes all decreased with NET. The positive changes of CiOQ increased significantly (Figure 3). Perceived social support and coping did not change as a result of the treatment.

## Discussion

In this study we examined the efficacy and safety of NET, a short-term treatment approach for therapy of traumatised adults. The results supported the efficacy of NET in treating adult survivors of the Sichuan earthquake. Significant effects were found across a number of psychological variables post treatment. Levels of reported symptoms of PTSD, depression and anxiety, and general mental health were significantly reduced, and these reductions were stable for the 2 month follow up. Negative posttraumatic changes were reduced and positive changes increased. NET had little effect on either coping styles or social support.

Significant improvements in posttraumatic symptom categories after NET may reflect the mechanism of emotional habituation elicited by the exposure [18] and the efficiency of the narrative approach in the remediation of distortion of the explicit autobiographic memory about traumatic events, such as intrusive memory fragment, avoidance of thoughts, and trauma reminders [16]. Depression, anxiety and general mental distress scores were also significantly reduced with NET. The impact of NET on reducing depression has been observed by Bichescu et al. [15]. This may because persistent posttraumatic symptoms contribute to additional psychological and physical disturbances [42,43]. It indicates that NET could reduce comorbid symptoms beyond the core set of PTSD.

The size of the treatment effect on posttraumatic symptoms at posttest (1.09-1.35) was higher than the effect sizes (0.6) reported in previous NET with traumatized refugees populations (e.g. [6]). The score on positive changes was relatively high in this sample (25-26/30), compared with other samples assessed using the CiOQ (20.50/30) [33]. Anecdotally, the participants generally reported a high positive appraisal of the government’s rapid response to the earthquake. The social and political context, in this case the positive view of the government, could have an influence on the outcome of psychotherapies, though this is speculative, and needs further systematic research.

The difference to previous NET studies may be ascribed to the Chinese state-led support and assistance after the earthquake, as opposed to the insecurity and severe economical problems with refugees.

In addition, an improvement in positive change (posttraumatic growth) and reduction in negative change after treatment was found, with the outcome being stable at the 2 month follow-up. This suggests that treatment not only decreases symptoms but may also improve growth. It is consistent with one study with 65 PTSD patients treated with exposure therapy which showed that people experienced posttraumatic growth through finding new possibilities and personal strengths [44]. The current findings may be linked to NET treatment focusing on helping people develop narratives about what has happened to them. Previous research has indicated the importance of narrative development for meaning-making after traumatic events [45].

Contrary to our hypothesis is there is only weak indication of improvement in perceived social support and coping. Other studies have found that chronic disorders such as PTSD can corrode social support [46,47]. However, NET did not specifically address how people can change their social behaviour; it may be too short to have a significant effect on social support. Regarding coping, findings in this area are mixed. Individual differences in coping style have been found to influence the transition from distress to disorder [48]. Other studies indicate that coping have limited predictive power for PTSD [23]. The relationship between events, coping and disorder is likely to be complicated [49]. Previous NET studies have not assessed coping style and little is known about the effect on coping of the intervention. However, this result may manifest that a short term intervention can unlikely change the coping. Alternatively, it may be that the measure chosen was not sensitive enough to pick up subtle changes. In addition, it may relate to coping style being a personality characteristic, and it is unlikely that a procedure such as NET will change its levels, or two month is too short to find the improvement.

The study was the first time NET has been applied to a Chinese setting and in earthquake-related PTSD. Although the efficacy of NET has already been shown across cultures in Europe, Africa and Asia [6,15,20], the psychosocial environment in this study was different from previous work which has largely focused on people affected by war and torture. While the choice of IES-R in this study was based on evidence of its effectiveness in other studies, a direct comparison of PTSD severity with previous NET studies using other measures (e.g. CAPS) is not possible. However, compared with populations of previous NET studies who experienced multiple or chronic traumatic events, particularly those originating from organised violence or torture of a severe and chronic nature, it is possible that earthquake survivors have a less severe or complex level of trauma. NET was originally designed to examine traumatic situations where there was a perpetrator, as it is derived in part from testimony therapy, which enables a witness document to be created. Nevertheless, the current study showed that for possibly simpler traumatic events without a perpetrator, NET can be effective. Further research is required to determine the effectiveness of NET with different types and severity of trauma. What this study indicates is the possibility of extending the approach outside of situations where testimony may be required and that people want to create narratives under different types of situations.

The lack of dropouts in the NET is in line with other NET studies [6,42]. Most participants informally reported that they felt relieved and more comfortable after NET. This may be because of the nature of the intervention. NET uses narrative, which is the approach we all use in interactions so, unlike approaches such as CBT, it has good face validity and is not intimidating to the participant.

The main limitation of the study is the sample size and the lack of a longer term follow up. The sample size is small as the study aimed to test the effectiveness of NET in a new population (Chinese, and no perpetrator of the traumatic event), and practical considerations meant that a longer term follow up was impractical, as many of the participants were being moved into new accommodation in the period after the study, and would not always be traceable. Clearly, the sample may not be representative of Chinese earthquake survivors in general – at least in part because most participants were women. This reflected the sampling typically found in research of this area [50] and in survivor population as and most men were out for work in day time. However, the overall effectiveness of the intervention demonstrated its utility in such circumstances.

We found that some participants were not interested in signing off their final written biography. It may because of their poor education level or – perhaps more likely – that there are no perpetrators so there is no need for a signed witness report. Furthermore, according to feedback, four sessions was too long for many participants. This was because it usually took one session on the narration of their previous trauma experiences of traumatic life events, but 10 of the 22 participants had no other trauma experiences except the earthquake. Therefore, most changes to the narrative and to symptoms appeared to occur in the first two sessions (though there is no empirical evidence for this, so it will be necessary to conduct further research to test this). Some participants reported automatically reduced PTSD symptoms (e.g. better sleep, less intrusion etc.) and improved well-being (e.g. starting to hum while walking) after two sessions. This may suggest the standard four-session NET could perhaps be adapted and shortened for disaster-related traumatic events. Studies with larger sample sizes are needed to investigate such matters and to extend the present findings. Our study provided evidence for the applicability of a western developed approach in the Chinese population.

## Conclusions

In conclusion, the NET appears to be an effective treatment for earthquake survivors displaying psychological symptoms. Not only is it effective for PTSD symptoms, but also for anxiety, depression and general mental health. NET, though initially designed for use with refugees and other victims of war, may be an effective treatment programme for a wider range of traumatic and stressful events. The benefits of NET are clear. What is needed is to determine its efficacy across different traumatised groups, and also to highlight the most effective ways of training people to administer it.

## Abbreviations

NET: Narrative Exposure Therapy; PTSD: Post Traumatic Stress Disorder; PTG: Posttraumatic Growth; IES-R: Impact of Event Scale-Revised; HADS: Hospital Anxiety and Depression Scale; CiOQ: Changes in Outlook Questionnaire; SCSQ: The Simplified Coping Style Questionnaire; GHQ-28: The General Health Questionnaire-28; MSPSS: The Multidimensional Scale of Perceived Social Support.

## Competing interests

The authors declare that they have no competing interests'.

## Authors' contributions

YZ designed the study, collected and analysed the data, and drafted the paper. NH designed the study, contributed to data analysis and paper writing. TC contributed to the data analysis and paper writing. All authors read and approved the final manuscript.

## Pre-publication history

The pre-publication history for this paper can be accessed here:

http://www.biomedcentral.com/1471-244X/13/41/prepub
